# The impact of local government investment on the carbon emissions reduction effect: An empirical analysis of panel data from 30 provinces and municipalities in China

**DOI:** 10.1371/journal.pone.0180946

**Published:** 2017-07-20

**Authors:** Lingyun He, Fang Yin, Zhangqi Zhong, Zhihua Ding

**Affiliations:** 1 School of Management, China University of Mining and Technology, Xuzhou, China; 2 School of Economics, Zhejiang University of Finance & Economics, Hangzhou, China; Nankai University, CHINA

## Abstract

Among studies of the factors that influence carbon emissions and related regulations, economic aggregates, industrial structures, energy structures, population levels, and energy prices have been extensively explored, whereas studies from the perspective of fiscal leverage, particularly of local government investment (*LGI*), are rare. Of the limited number of studies on the effect of *LGI* on carbon emissions, most focus on its direct effect. Few studies consider regulatory effects, and there is a lack of emphasis on local areas. Using a cointegration test, a panel data model and clustering analysis based on Chinese data between 2000 and 2013, this study measures the direct role of *LGI* in carbon dioxide (*CO*_*2*_) emissions reduction. First, overall, within the sample time period, a 1% increase in *LGI* inhibits carbon emissions by 0.8906% and 0.5851% through its influence on the industrial structure and energy efficiency, respectively, with the industrial structure path playing a greater role than the efficiency path. Second, carbon emissions to some extent exhibit inertia. The previous year’s carbon emissions impact the following year’s carbon emissions by 0.5375%. Thus, if a reduction in carbon emissions in the previous year has a positive effect, then the carbon emissions reduction effect generated by *LGI* in the following year will be magnified. Third, *LGI* can effectively reduce carbon emissions, but there are significant regional differences in its impact. For example, in some provinces, such as Sichuan and Anhui, economic growth has not been decoupled from carbon emissions. Fourth, the carbon emissions reduction effect in the 30 provinces and municipalities sampled in this study can be classified into five categories—strong, relatively strong, medium, relatively weak and weak—based on the degree of local governments’ regulation of carbon emissions. The carbon emissions reduction effect of *LGI* is significant in the western and central regions of China but not in the eastern and northeast regions. This study helps overcome the limitations of previous studies on the regulatory effects of *LGI* on carbon emissions, and the constructed model may more closely reflect actual economic conditions. Moreover, the current study can benefit countries similar to China that aim to objectively identify the impacts of their *LGI* on carbon emissions, and such countries can use it as a reference in the formulation of investment policies based on their economic and industrial characteristics.

## Introduction

Since the Intergovernmental Panel on Climate Change (IPCC) released its third climate evaluation report, climate warming has gradually become an increasingly important agenda item. Countries must take on the important tasks of developing low-carbon economies and addressing climate warming. The Paris agreement, which was approved on December 12, 2015, sets the following goal: “To achieve a balance between anthropogenic emissions and removals of greenhouse gases in the second half of this century”.

China ranks first in carbon emissions in the world and has committed itself to conserving energy and reducing emissions. Presently, it has entered a “new normal” stage of economic development that provides opportunities for energy conservation and emissions reduction. On the one hand, economic growth will slow; as a result, the demand for energy will decrease. *China’s Energy Prospects in 2030* forecasts that in the context of the energy revolution, the growth of China’s total energy demand will slow, reaching 4.8 and 5.3 billion tons of standard coal equivalents in 2020 and 2030, respectively, and that carbon emissions will peak in 2025. On the other hand, structural reform will be a core issue for China’s supply side, particularly regarding its industrial structure. Improving the country’s energy efficiency is the focus of China’s supply-side energy reforms, and all of these components will be helpful for realizing the goals of energy conservation and emissions reduction. Thus, China is creating a structural system that includes essential general policies and other supplementary instruments to encourage energy conservation and emissions reduction. The general policies mainly refer to prices and fiscal leverage. On August 22, 2012, the Twelfth Five-year Plan for Energy Conservation and Emissions Reduction was released. This document noted that China still faces challenges with regard to economic policy imperfections in areas such as prices, finance and taxation, and it emphasized the need to accelerate reforms of the supply-side fiscal and taxation systems. However, until now, studies on the effect of fiscal leverage on carbon emissions reduction have been rare. Furthermore, even among the small number of relevant studies, most have focused on taxation policies and the effects of fiscal policy; local government investment (*LGI*), in particular, is seldom considered.

Thus, this study explores the paths of influence of *LGI* on carbon emissions and attempts to determine their effects. Regional differences in effects are also explored. The results of this study can benefit the objective localization of the government’s energy conservation and emissions reduction policies. It can also provide a basis for local governments to formulate effective energy conservation and emissions reduction policies based on their respective economic situations. This study is organized as follows: In the second section, we briefly review the relevant literature. In the third section, the theoretical association between local governments and carbon emissions and the paths of influence for local governments on carbon emissions are analyzed. The fourth section develops the model and describes the data used. The fifth section presents the evaluation outcomes based on the constructed model. The sixth section focuses on regional differences in the effects of *LGI* on carbon emissions reduction, and the last section provides the study’s conclusions.

## Literature review

From a research perspective, the key to reducing carbon emissions is identifying the major factors that influence them, including both productive and consumptive factors. The research methods used include both factor decomposition and other empirical methods. The former mainly refers to the log mean Divisia index (LMDI) method and the Kaya identity in order to decompose the influencing factors, while the latter measure the relationships between various factors and carbon emissions based on theory. Research shows that the level of economic development, the economic structure, energy intensity, technological progress, the urbanization level, the degree of trade openness, population and other factors all impact carbon dioxide (*CO*_2_) emissions [1–14]. From the perspective of this study, two of these factors merit attention. First, we consider the relationship between the level of investment and carbon emissions. Many scholars in China and elsewhere acknowledge this linkage and contend that investment has a pulling effect on carbon emissions. Most of these studies focus on foreign direct investment (*FDI*) and fixed asset investment (*FAI*) [15, 16]. Some scholars have noted a decoupling of the relationship between economic growth induced by investment and carbon emissions [17]. Others argue that China can potentially mitigate its carbon emissions through domestic investment [18, 19]. Second, we consider the relation between structural and efficiency (technical) factors and carbon emissions. Many studies have confirmed the impact of these two factors [20–23]. However, for the structural and efficiency paths to function, they require the advancement of a comprehensive set of policy tools. Therefore, many scholars also examine the relationships between policy tools and carbon emissions; the majority of them focus on prices and taxation. Most of the research from a financial perspective affirms the relations among energy prices, energy consumption and carbon emissions, arguing that reasonable energy prices can effectively lower carbon emissions [24–27]. From the perspective of finance and taxation, most of the research focuses on taxation, with few studies considering fiscal expenditures. Drezner notes that tax preferences influence the direction in which future energy policy develops [28]. Glomm et al. and Hubler argue that government taxation is instrumental in motivating enterprises and society to implement energy conservation measures and reduce emissions [29, 30]. An increase in taxation can reduce carbon emissions to a certain extent. Meanwhile, fiscal expenditures comprise fiscal investments, fiscal subsidies, revenues and expenditures of extra-budgetary funds. This study focuses on fiscal investment policy.

Fiscal investment refers to the investment behaviors adopted by central and local governments using funds accumulated by fiscal means or through raised funds. From the perspective of fiscal investment, Purohit and Purohit claim that fiscal investment plays a significant role in mitigating emissions [31]. Some research focuses on emissions reduction through different types of fiscal investments. Mahesh and Jasmin suggest that expanding investment in renewable energy sources on a large scale can effectively reduce emissions and help create a sustainable, low-carbon economy [32]. Framstad and Strand and Zhang find that investment in infrastructure can impact carbon emissions [33, 34]. Lee et al. conclude that investment in environmental protection facilitates a low-carbon industrial structure and is helpful in reducing emissions [35]. On the whole, the research from the fiscal investment perspective analyzes general components. In China’s unique economic system, energy conservation and emissions reduction policies are guided by the central government, but local governments’ economic behaviors are key influences on such efforts [36]. Therefore, several Chinese scholars have recently begun to examine the relationship between *LGI* and carbon emissions. Zhang notes that an increase in *LGI* can reduce the emissions of various pollutants and improve the environment [37]. Peng uses a STIRPAT model in an empirical analysis that shows how *LGI* helps to reduce carbon emissions [38]. Zhang et al. conclude that the influence of *LGI* bias on *CO*_*2*_ bias is significant and positive [17]. In addition, *LGI* has inverted U-shaped effects on *CO*_*2*_ emissions. In the initial stage, *LGI* aggravates *CO*_*2*_ emissions. Thus, with further increases in investment, emissions first deteriorate and then start to improve. However, fiscal investment does not have a direct impact on carbon emissions. Instead, it influences carbon emissions through a series of intermediate variables, such as economic aggregates, the industrial structure and energy efficiency (technology). In terms of the relationships between *LGI* and economic aggregates, most scholars recognize that *LGI* has a pulling effect on the economy [39]. However, some researchers emphasize the regional differences in this effect [40]. From a structural perspective, Song and Wang note that *LGI* can effectively optimize the industrial structure, while Peng, using an empirical test of panel data, concludes that government investment in energy-related industries significantly decreases energy intensity (*Energy efficiency* = 1/*Energy intensity*, namely, a decrease in energy intensity means an increase in energy efficiency.) [41, 42].

In summary, scholars have focused on the impact of government investment on carbon emissions, thus laying the foundation for subsequent studies. However, the previous studies suffer from some limitations. First, under an energy conservation and emissions reduction model directed by the central government, the economic behaviors of local governments are essential to achieving the policy goals. However, studies focusing on *LGI* are rare. Second, the variables (including both productive and consumptive variables) that influence carbon emissions are simultaneously introduced into the models. Thus, the focus is on the direct role of *LGI* in carbon emissions, without in-depth investigations of its paths of influence or its regulating effect via these paths. Third, China has a unique policy environment in which considerable regional differences in economic development and policy application exist. However, the extant studies lack analyses and comparisons of the regional differences in the impacts of *LGI* on carbon emissions. In terms of Chinese practices, on the one hand, local governments are important participants in the development of China’s low-carbon economy, playing major roles in energy conservation and emissions reduction. On the other hand, China’s economic development exhibits regional imbalances. From the perspective of *LGI*, this study examines local governments’ direct and regulatory roles in carbon emissions policy and further analyzes regional differences. This study may be significant in the exploratory construction of regulating effect models and with regard to regional divisions based on the effects of *LGI* on emissions reduction.

## Theoretical foundations for the carbon emissions reduction effect of LGI

### Correlation between LGI and carbon emissions

The premise for examining the impact of *LGI* on carbon emissions reduction is that *LGI* can in fact influence the level of carbon emissions. Consistent with the cardinal principles of economics, this study follows the research of the Chinese scholars Zhang et al. [17]. Their study supposes that there are no differences among individuals in an economy, and the effect of a representative individual at time *t U*(⋅) is dependent on the consumption level *C*_*t*_ and the environmental level *S*_*t*_. Expression (1) is derived as follows [17]:

$$W_{E}=C_{t}+D_{t}+E_{t}+\frac{U_{S}}{U_{C}}S_{0}-\tau e_{t}$$ (1)

where *W*_*E*_ is the overall social welfare, *D*_*t*_ is the level of productive investment, *E*_*t*_ is the level of investment in emissions reduction, *S*_*0*_ is the environmental level under the original conditions, *e*_*t*_ is carbon dioxide emissions, *S*_*t*_ = *S*_*0*_−*ae*_*t*_, and *U*_*S*_ and *U*_*C*_ are the partial derivatives of the utility function *U*(⋅) with respect to the environmental level *S*_*t*_ and consumption *C*_*t*_, respectively. Here, $\frac{U_{S}}{U_{C}}$ can be taken as the consumption-measured price that a utility-maximizing consumer is willing to pay per unit of the marginal environmental level. According to the above expressions, the current carbon emissions level reduces the overall economic welfare (as measured by marginal emissions reduction costs or marginal social costs), while *D*_*t*_ and *E*_*t*_ are forces that can balance emissions and decrease the economic benefits. In the initial stage, local governments make sufficient investments in production but insufficient investments in emissions reduction; as a result, carbon emissions gradually increase. As long as the pulling effect of productive investments on welfare exceeds the emissions effect, social economic benefits are enhanced. Once the social welfare is improved, it is unlikely to decline, so emissions gradually grow. Therefore, local governments increase their investment to reduce emissions and ameliorate any downward pressure on the local welfare. *e*_*t*_ is a decreasing function of *E*_*t*_ [17]. Therefore, the increase in emissions at this stage flattens or even decreases. In a later stage of development, due to the transformation of the development mode, carbon emissions gradually decline with increases in *LGI*. Therefore, in theory, *LGI* and carbon emissions show an inverted U-shaped relationship.

The above analysis highlights three points. First, in theory, *LGI* can impact carbon emissions. Second, the role played by *LGI* depends heavily on its investment structure. Third, the impact of *LGI* on carbon emissions is a result of the combined actions of various factors.

### Influencing paths of LGI’s effect on carbon emissions

Based on the available studies, it is clear that *LGI* can impact carbon emissions. However, in actual economic operations, this impact is more complex than it seems. The impact of *LGI* is realized through various paths. A review of studies by Chinese and other scholars shows that *LGI* can affect economic aggregates, the industrial structure and energy efficiency, among which the structural and efficiency (technical) paths are most closely related to emissions reduction. Peng notes that *LGI* has an effect on carbon emissions mainly through optimization and upgrading of the industrial structure and by directly supporting energy conservation and emissions reduction projects [38]. In fact, on the one hand, by guiding related industrial investment and policy, *LGI* helps enterprises make use of new technologies to transform traditional industries, improve energy efficiency and reduce environmental pollution. On the other hand, through market operations, *LGI* raises the prices of investment goods and production materials, resulting in changes to production costs. Production costs and market supply and demand further affect the industrial structure and energy efficiency. This paper examines *LGI*’s direct role and regulating role in carbon emissions through the structure and efficiency paths. In addition, by considering regional diversity in economic development, industrial characteristics and local government behavior, this study compares the correlations between *LGI* and carbon emissions in 30 provinces and municipalities (henceforth, collectively described as provinces) in China and classifies them into different categories based on *LGI*’s role in reducing emissions.

## Models, variables and data

### Modeling

Based on the above analysis, this study emphasizes two points. First, *LGI* cannot affect carbon emissions directly. Instead, it influences carbon emissions through various paths, namely, “*LGI*–intermediate variables–carbon emissions”. This study primarily investigates the emissions reduction effect of *LGI*, which is mainly achieved through the structure and efficiency paths. Second, this study “black boxes” the “*LGI*–intermediate variables” process. First, the apparent emissions reduction effect is examined. Then, the “black box” is opened to investigate the comprehensive regulating role of *LGI* on carbon emissions through the structure and efficiency paths.

According to Kaya, the factors influencing carbon emissions can be categorized as carbon emissions intensity, energy intensity, per capita *GDP* and population, as in Expression (2) [43]:

$$CO_{2}=\frac{CO_{2}}{E_{c}}\times \frac{E_{c}}{GDP}\times \frac{GDP}{P}\times P$$ (2)

where *CO*_*2*_ is carbon dioxide emissions; *E*_*c*_ is the total energy consumed; and $\frac{CO_{2}}{E_{c}}$ $\frac{CO_{2}}{E_{c}}$, $\frac{GDP}{P}$, and *P* represent carbon emissions intensity, energy intensity, per capita *GDP* and population, respectively. Furthermore, carbon emissions intensity is influenced by the energy consumption structure and a carbon emissions coefficient:

$$\frac{CO_{2}}{E_{C}}=\frac{E_{ci}}{E_{c}}\times \frac{E_{c}}{CO_{2i}}$$ (3)

where *E*_*ci*_ is the consumption of the *i*th type of energy, and $\frac{E_{ci}}{E_{c}}$ and $\frac{E_{c}}{CO_{2i}}$ are the energy consumption structure and *CO*_*2*_ emissions coefficients, respectively. Therefore, carbon emissions are determined by the energy consumption structure, the *CO*_*2*_ emissions coefficient, energy intensity, per capita *GDP* and population. These factors can be classified as productive and consumptive factors, whereby population is a consumptive factor, and the rest are productive factors. With regard to these factors, first, in this study, carbon emissions are considered from the perspective of productive factors; therefore, the impact of population on carbon emissions is not considered in this study. Second, per capita *GDP* is an indicator that reflects the economic scale. The current study investigates the relation between LGI and carbon emissions, where the total amount of *LGI*, in terms of the variable’s attributes, is also measured on a scale indicator. Moreover, since the implementation of China’s fiscal decentralization reforms in 1994, *LGI* spending has accounted for a constantly increasing proportion of local *GDP* [44]. Given that this study begins from the production perspective, per capita *LGI* has no practical economic significance; therefore, based on the above analysis, this study uses *LGI* in place of per capita *GDP* in the Kaya identity. Third, under the given technical conditions, the *CO*_*2*_ emissions coefficient is constant over a short period and is therefore not considered. Fourth, given the current conditions, it is difficult for China’s energy consumption structure, which is reliant on coal, to change rapidly within a short period. However, the industrial structure can exert an important influence on energy consumption. Meanwhile, due to different industrial characteristics, the demand for different types of energy can also differ across industries, which can further impact the energy structure [45, 46]. Therefore, the industrial structure is used in place of the energy structure. Fifth, energy intensity reflects the state of energy use efficiency. Given the theoretical analysis in this study, an energy efficiency indicator is used in place of energy intensity. Therefore, Expression (4) is derived as follows:

$$CO_{2}=Inv\times S\times E$$ (4)

where *Inv* is *LGI*, *S* is the industrial structure, and *E* is energy efficiency. A panel data model for 30 provinces in China is constructed in this study. To eliminate heteroscedasticity and derive the elasticity directly, related variables are expressed as natural logarithms, leading to Expression (5):

$$LnCO_{2it}=C_{i}+\alpha LnInv_{it}+\beta LnS_{it}+\gamma LnE_{it}+\varepsilon_{it}$$ (5)

Expression (5) is the direct effect model, in which *i* represents provinces (*i* = 1, 2, …, 30, which are Beijing (BJ), Tianjin (TJ), Hebei (HB), Shanxi (SX), Inner Mongolia (NMG), Liaoning (LN), Jilin (JL), Heilongjiang (HLJ), Shanghai (SH), Jiangsu (JS), Zhejiang (ZJ), Anhui (AH), Fujian (FJ), Jiangxi (JX), Shandong (SD), Henan (HN), Hubei (HUBEI), Hunan (HUNAN), Guangdong (GD), Guangxi (GX), Hainan (HAINAN), Chongqing (CQ), Sichuan (SC), Guizhou (GZ), Yunnan (YN), Shaanxi (SHANXI), Gansu (GS), Qinghai (QH), Ningxia (NX) and Xinjiang (XJ)). Due to data inaccessibility, Tibet, Taiwan, Hong Kong and Macao are not taken into consideration. Here, t denotes the year, and *C*_*i*_ indicates the intercept terms in the ith cross-member equations (including fixed and random effects), which reflect the individual influences, that is, they reflect the neglected influence of cross-differences in the model, and *ε*_*it*_ is the random disturbance variable. Furthermore, according to the analysis in Sections 1 and 2 of this paper, *LGI* exerts a regulating role mainly through the structure and efficiency paths. To reflect this type of indirect impact, this study, drawing on the work of Chen et al., first combines basic principles of econometrics and adopts interaction terms to represent the regulating role [47]. Second, according to Zhou et al. [48], technical progress itself does not reduce emissions but indirectly leads to decreasing emissions by upgrading and optimizing the industrial structure. Therefore, this guiding role is incorporated into the model. Third, given the apparent inertia of each province’s carbon emissions mechanism, previous carbon emissions have a notably positive influence on current emissions [49]. Therefore, previous carbon emissions are also incorporated into the model to derive a dynamic regulating effect model, as in Expression (6):

$$LnCO_{2it}=C_{i}+\alpha_{i}Ln{\left(Inv\times S\right)}_{it}+\beta_{i}Ln{\left(Inv\times E\right)}_{it}+\gamma_{i}Ln{\left(S\times E\right)}_{it}+\eta_{i}LnCO_{2it-1}+\varepsilon_{it}$$ (6)

### Variables and data

The study interval is between 2000 and 2013, and the main variables are selected as follows:

(1) carbon emissions *CO*_*2*_. This study refers to the formulae and the default value of the carbon emissions coefficient based on guidance provided by the IPCC and directly measures *CO*_*2*_ emissions based on consumption data for different types of fossil fuels, as indicated in Expression (7):

$$CO_{2i}=\sum_{j=1}^{8}Q_{ij}\times C_{j}$$ (7)

where *Q*_*ij*_ is the jth type of energy consumption in the ith province, expressed as standard coal equivalents. The data come from the China Energy Statistical Yearbook and the China Statistical Yearbook and are reported in units of ten thousand tons [50, 51].

(2) local Government investment *Inv*. Government investment refers to the phenomenon in which the government, a special investor, makes investments in specific areas via fiscal expenditures to promote the coordinated development of different sectors of the national economy and ultimately foster socioeconomic development. However, the China Finance Yearbooks do not provide an indicator for *LGI*. Therefore, based on the definition given by Zhang [40] and using the function-based fiscal expenditures classification from the China Finance Yearbooks (i.e., the *LGI* that is mainly used for culture and education, agriculture, administration, and social security assistance), *LGI* is proxied by the share of science, education, culture, health and administrative expenditures of total fiscal expenditures in a local government’s common budget. [51]. Administrative expenses during the 2007–2013 period are not listed here. Related expenditures for Fujian Province include Xiamen City during the 2000–2004 period and do not include Xiamen after 2004. In addition, the educational expenditures data for Tanjin Municipality are missing for 2002, so they are estimated using an educational expenditures regression for Tianjin during the 2000–2013 period (a growth curve is selected for the estimation). The data come from the China Finance Yearbook in units of hundred million yuan.

**Fig 1:**
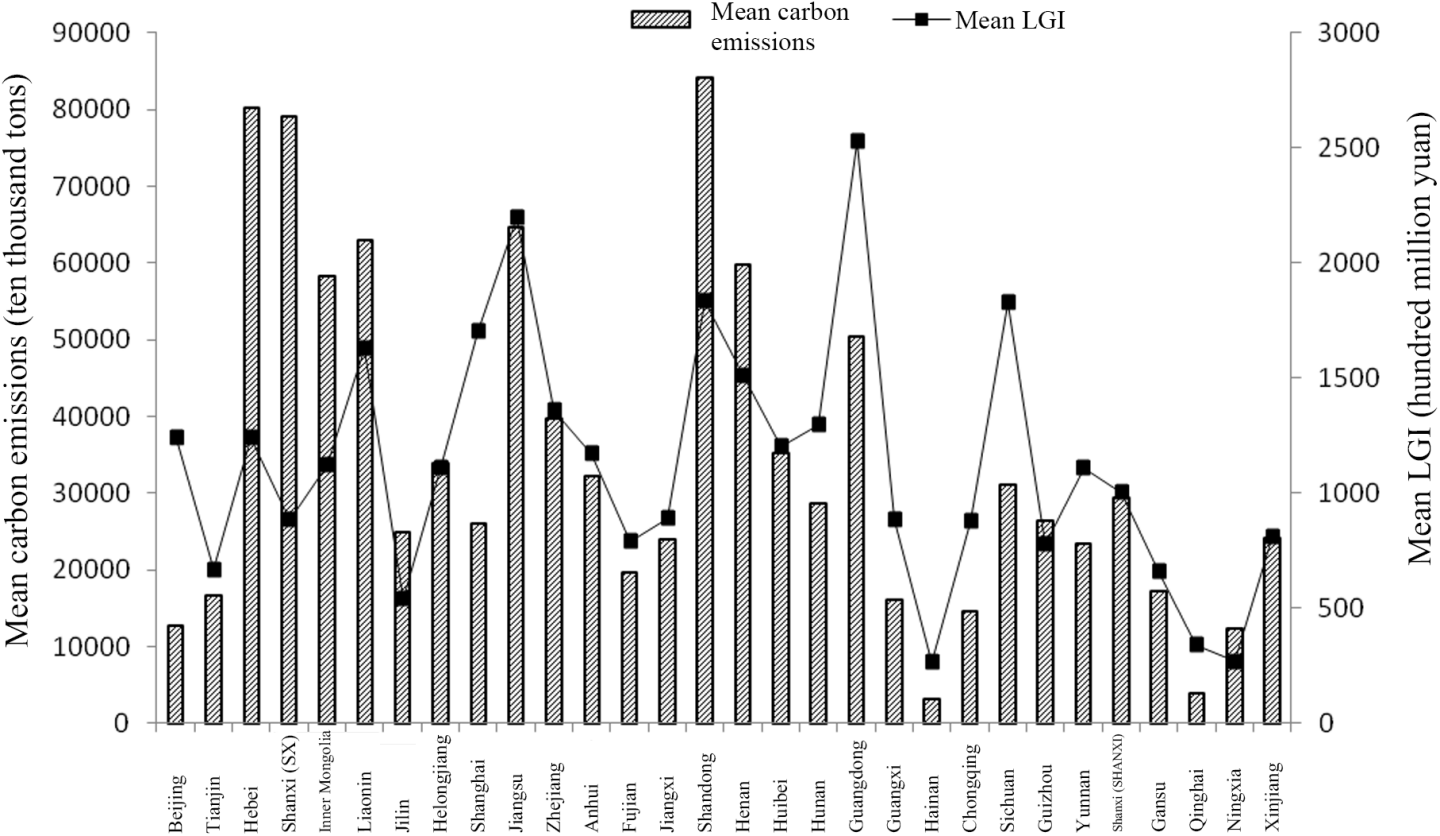
Mean *LGI* and carbon emissions in 30 provinces in China. Calculations are based on data from the China Finance Yearbook (2001–2014), the China Energy Statistical Yearbook (2013) and the China Statistical Yearbook (2001–2014).

The mean carbon emissions of a sampled province during the 2000–2013 period represent the overall level of carbon emissions in that provincial area during that period. Similarly, the mean *LGI* of each province during the 2000–2013 period represents the overall level of *LGI* in each provincial area. The variation in *LGI* and carbon emissions for the 30 sampled provinces is shown in Fig 1.

In Fig 1, the variation in mean carbon emissions has no rigid correspondence with the variation in mean *LGI*. Total *LGI* is rather high in Hebei, Inner Mongolia, Liaoning, Shanghai, Shandong, Guangdong and Sichuan, while it is rather low in Jilin, Hainan, Qinghai and Ningxia. Provinces with rather large mean carbon emissions include Hebei, Shanxi, Inner Mongolia, Liaoning, Jiangsu, Shandong, Hainan, Hubei and Guangdong, while the provinces with rather small mean carbon emissions include Beijing, Tianjin, Fujian, Guangxi, Hainan, Gansu, Qinghai, Ningxia and Xinjiang. For most provinces, a higher *LGI* corresponds to relatively high carbon emissions. Provinces with low carbon emissions have significantly lower *LGI*. Several provinces have relatively high mean *LGI* but relatively low carbon emissions. However, a variety of factors influence carbon emissions, and there are also regional differences in emissions. In addition, Fig 1 shows the mean *LGI* and carbon emissions as absolute amounts rather than as growth rates; therefore, it is difficult to determine the actual impact of *LGI* on carbon emissions from the figure.

(3) industrial structure *S*. *GDP* is calculated based on the production method adopted by the National Bureau of Statistics of the PRC and combined with the value added of secondary industry as a share of *GDP*.

(4) energy efficiency *E*. This study draws on the definition of energy efficiency proposed by Wang et al. [52], which uses the reciprocal of energy intensity, the “unit energy consumption of *GDP*” (*GDP*/total energy consumption), as an indicator of overall energy efficiency in units of hundred million yuan/ten thousand tons of standard coal equivalents. These data come from the China Energy Statistical Yearbook and the China Statistical Yearbook [53, 54].

## Model estimation and analysis of results

### Direct effect model estimation

Before the model estimation, a stationarity test is applied to the related sequences. This paper adopts the Augmented Dickey-Fuller (ADF) method for unit root testing of panel data, and the results indicate that the tested sequences are all I(1) sequences, which can be studied further. To avoid spurious regression results, related variables are tested for cointegration. Table 1 shows the results of the Johansen cointegration tests.

**Table 1:** Results of Johansen cointegration tests.

Variable	Null hypothesis	Trace test	p-value
*LnCO*_*2*_ and *LnInv*	No cointegration	229.0	0.0000*
	One cointegration at most	146.8	0.0000*
*LnCO*_*2*_ and *LnS*	No cointegration	164.2	0.0000*
	One cointegration at most	120.5	0.0000*
*LnCO*_*2*_ and *LnE*	No cointegration	176.8	0.0000*
	One cointegration at most	92.16	0.0000*

* indicates that the null hypothesis is rejected at the 1% confidence level.

The test results show that a long-term equilibrium relationship exists among *LGI*, industrial structure and energy efficiency at the 1% significance level. Further, based on the results of a Hausman test (the Chi-Sq value is 35.9069 and the null hypothesis is rejected), a fixed effect model should be estimated. To demonstrate the reliability of the fixed effect model, cross-sectional dependence tests were performed. The p-values according to the Pesaran test and the Friedman test are 0.0000 and 0.0196, respectively, rejecting the original hypothesis at the 95% confidence level, which indicates the existence of a cross-sectional dependence problem. However, at the 99% confidence level, the Friedman test denies the existence of a cross-sectional dependence problem, indicating that the fixed effect model is reliable. A least squares (LS) regression method is adopted to estimate model (5). The results are shown in Table 2.

**Table 2:** LS estimation results of the direct effect model.

Variable	Coefficient	Standard deviation		t statistic	p-value
*C*	6.1136	0.2415		25.3112	0.0000
*LnS*	0.5957	0.1011		5.8907	0.0000
*LnE*	-0.8253	0.0730		-11.3068	0.0000
*LnInv*	0.6591	0.0267		24.7186	0.0000
*C*_*1*_	-0.0884	*C*_*11*_	0.4569	*C*_*21*_	-0.6716
C_2_	-0.0817	C_12_	0.2512	C_22_	-0.4299
C_3_	0.9604	*C*_*13*_	0.1337	*C*_*23*_	-0.2317
*C*_*4*_	0.4897	*C*_*14*_	0.2088	*C*_*24*_	-0.2766
*C*_*5*_	0.2576	*C*_*15*_	0.7329	*C*_*25*_	-0.3061
*C*_*6*_	0.3088	C_16_	0.4822	*C*_*26*_	0.0107
*C*_*7*_	-0.0295	*C*_*17*_	0.1791	*C*_*27*_	-0.4724
*C*_*8*_	0.0201	*C*_*18*_	0.0377	*C*_*28*_	-1.8116
*C*_*9*_	0.0577	*C*_*19*_	0.3762	*C*_*29*_	-0.6579
*C*_*10*_	0.6511	*C*_*20*_	-0.2317	*C*_*30*_	-0.3447
*R*^*2*^		0.9764	*F* statistic		495.6244
Adjusted *R*^*2*^		0.9744	Mean of dependent variable		10.1433
Regressed standard deviation		0.1404	Variance of dependent variable		0.8776
Residual sum of squares		7.5743	Durbin-Watson statistic		0.7084

*C*_*1*_, *C*_*2*_,…, *C*_*30*_ are the intercept terms in Eq 5, which correspond to the thirty provinces/cities.

From Table 2, we can conclude the following:

(1) When all variables function jointly, for every 1% increase in energy efficiency and every 1% increased optimization of the industrial structure, carbon emissions are inhibited by 0.8253% and 0.5957%, respectively, thus indicating that to a certain extent and within the sample time period, increased energy efficiency and optimization of the industrial structure can effectively reduce emissions. Our results are consistent with the literature [55, 56]. Meanwhile, our results indicate that the efficiency path has a greater effect than the structural path, which supports the conclusion drawn by He et al. [57]. Nevertheless, *LGI* does not directly cause carbon inhibition. Instead, it has a pulling effect on carbon emissions to a certain extent. Specifically, every 1% increase in *LGI* increases carbon emissions by 0.6591%, which seems inconsistent with reality. However, the theoretical analysis shows that *LGI* and carbon emissions exhibit an inverted U-shaped relationship [17], indicating that the overall relation between *LGI* and carbon emissions in China is still on the left side of the inverted U-shaped curve, having not yet achieved carbon decoupling. The direct effect model reveals a positive impact of *LGI* on carbon emissions and does not deny a carbon inhibition effect of *LGI*, which is consistent with Peng’s conclusion that “at the present stage, the investment of local government contributes to reduction of carbon emissions” [38]. The reasons are as follows: first, variation in carbon emissions is a result of the comprehensive actions of many variables, including the interplay among “economic pulling”, “economic inhibition”, “carbon pulling” and “carbon inhibition”. Meanwhile, carbon emissions function as a comprehensive indicator, while *LGI* not only exerts an inhibitory role on carbon emissions but also has a pulling effect on carbon emissions through economic aggregates. These roles might mutually offset, meaning that *LGI*’s inhibition of carbon emissions is not fully manifested, as addressed by Zheng et al. [58]. Second, due to differences in natural geographic conditions, economic development levels and consumptive structures, the impact of *LGI* on carbon emissions differs across provinces. In some provinces, the relationship between the two might be on the left side of the inverted U-shaped curve, but it cannot be denied that the inhibition of carbon emissions by *LGI* has manifested to some extent in some provinces. Third, the inhibition of carbon emissions is realized through multiple paths, and the overall carbon pulling effect does not negate carbon inhibition via other paths.

(2) Of the 30 sampled provinces, *LGI* inhibits carbon emissions in Beijing, Tianjin, Jilin, Guangxi, Hainan, Chongqing, Sichuan, Guizhou, Yunnan, Gansu, Qinghai, Ningxia and Xinjiang. If such inhibition is viewed in terms of economic areas, with the exception of Beijing, Tianjin, Hainan and Guangxi, these provinces are located in the western China (According to the National Bureau of Statistics of the PRC in 2011, in terms of economic zone classifications, Chinese provinces and municipalities can be grouped into eastern, central, northeast and western areas, where the eastern area includes Beijing, Tianjin, Hebei, Shandong, Jiangsu, Shanghai, Zhejiang, Fujian, Guangdong and Hainan; the central area includes Shanxi, Henan, Hubei, Hunan, Jiangxi and Anhui; the northeast area includes Heilongjiang, Jilin and Liaoning; and the western area includes Chongqing, Sichuan, Guangxi, Guizhou, Yunnan, Shaanxi, Gansu, Inner Mongolia, Ningxia, Xinjiang, Qinghai and Tibet). In fact, economic development is relatively slow in the western area. However, China has introduced many preferential policies to support the development of western China. *LGI* is gradually increasing, which will facilitate technological innovation and structural optimization and result in effective inhibition of carbon emissions. In Beijing and Tianjin, where the levels of economic development and openness are relatively high, local governments make large investments in industrial structure adjustments and technological innovation, and the effect on the inhibition of carbon emissions is rather obvious. In Hainan, the State Council issued Opinions to Promote the Construction and Development of Hainan International Tourism Island (No.44 [2009] by the State Council) to encourage tertiary industry, which relies primarily on tourism, in order to optimize the industrial structure. Elsewhere, particularly in the central area, the pulling effect of carbon emissions is quite large. In fact, the central area is an important energy base, with sizable energy consumption and carbon emissions; therefore, other carbon pulling effects might offset the emissions reduction effect of the policy.

## The dynamic regulation effect model estimation

When estimating model (6), this study considers two cases in which the first-order lag of *CO*_*2*_ is either considered or not and then compares the two sets of results. The LS method is adopted for the estimation, and the results are presented in Table 3.

**Table 3:** LS estimation results of the regulating model.

Variable	Coefficient	Standard deviation	t statistic	p-value	
*C*	6.1136	0.2415	25.3112	0.0000	
*Ln*(*Inv*×*S*)	1.0400	0.0529	19.6746	0.0000	
*Ln*(*Inv*×*E*)	-0.3810	0.0499	-7.6393	0.0000	
*Ln*(*S*×*E*)	-0.4443	0.0845	-5.2567	0.0000	
*C*_*1*_	-0.0884	*C*_*11*_	0.4569	*C*_*21*_	-0.6716
C_2_	-0.0817	C_12_	0.2512	C_22_	-0.4299
C_3_	0.9604	*C*_*13*_	0.1337	*C*_*23*_	-0.2317
*C*_*4*_	0.4897	*C*_*14*_	0.2088	*C*_*24*_	-0.2766
*C*_*5*_	0.2576	*C*_*15*_	0.7329	*C*_*25*_	-0.3061
*C*_*6*_	0.3088	C_16_	0.4822	*C*_*26*_	0.0107
*C*_*7*_	-0.0295	*C*_*17*_	0.1791	*C*_*27*_	-0.4724
*C*_*8*_	0.0201	*C*_*18*_	0.0377	*C*_*28*_	-1.8116
*C*_*9*_	-0.0577	*C*_*19*_	0.3762	*C*_*29*_	-0.6579
*C*_*10*_	0.6511	*C*_*20*_	-0.2394	*C*_*30*_	-0.3447
*R*^*2*^	0.9747		*F* statistic	477.6775	
Adjusted *R*^*2*^	0.9726		Mean of dependent variable	10.1433	
Regressed standard deviation	0.1452		Variance of dependent variable	0.8776	
Residual sum of squares	8.1193		Durbin-Watson statistic	0.6337	

*C*_*1*_, *C*_*2*_,…, *C*_*30*_ are the intercept terms in Eq 5 corresponding to the thirty provinces/cities.

(1) Combining Tables 2 and 3, it can be observed that when considering the role of *LGI* in regulating the related path variables, the role of the structure and efficiency paths in carbon emissions is strengthened. Specifically, a 1% increase in *LGI* inhibits carbon emissions by 1.0400% by guiding the industrial structure, which increases by 0.4443% compared with carbon inhibition via industrial structure optimization in the direct effect model. This finding supports that of Zhang and strengthens the evidence that *LGI* plays a role in guiding the industrial structure toward optimization [39]. Moreover, *LGI* inhibits carbon emissions by 0.3810% by influencing energy efficiency, which decreases by 0.4443% compared with that via the efficiency path in the direct effect model. On the one hand, this result indicates that the guiding role of *LGI* is not yet fully displayed. On the other hand, the impact of *LGI* on energy efficiency is realized through technology, and it is very difficult to fully realize technological innovation over a short period. Thus, the guiding role of *LGI* in energy efficiency is not fully displayed. Furthermore, energy efficiency (technical factors) also inhibits carbon emissions by 0.4443% by guiding the industrial structure, which reflects that coordination among various paths can increase the carbon emissions reduction generated by *LGI*. Overall, the emissions reduction model established in this study indicates that the role of the structural path is stronger than that of the efficiency path.

(2) The regulating role of *LGI* differs across the sampled provinces in China through the structural and efficiency paths in accordance with the direct effect model. In some provinces, economic development has not yet achieved carbon decoupling, and *LGI* has not facilitated carbon emissions reduction, generating a carbon pulling effect instead. Nevertheless, *LGI* in other provinces has generated effective carbon inhibition. As noted in Table 3, *LGI* inhibits carbon emissions in Hainan, Ningxia, Gansu, Chongqing, Xinjiang, Yunnan, Guizhou, Guangxi, Sichuan, Beijing, Tianjin, Shanghai and Jilin. With the exception of Hainan, Beijing, Tianjin, Shanghai and Jilin, the other provinces are in the western area. Overall, *LGI* generates higher carbon inhibition in China’s western area than in the central and eastern areas. In the eastern area, only Jilin’s *LGI* enhances carbon emissions reduction. When the first-order lag is included, given the dynamic nature of the model, the generalized method of moments (GMM) proposed by Arellano and Bond is adopted for the 30 sampled provinces [59]. The results are shown in Table 4.

**Table 4:** GMM estimation results for the dynamic regulating model.

Region	*α*	*β*	*γ*	*η*	J statistic
BJ	0.9992(0.2420)	-0.5453(0.0856)	-0.0057(0.2106)	0.8306(0.1027)	0.2150
TJ	0.5319(0.1734)	-0.3470(0.1062)	-0.0572(0.0990)	0.9205(0.0462)	0.1458
HB	0.0535(0.1421)	-0.2001(0.1372)	0.4224(0.1721)	1.1238(0.0427)	0.1704
SX	0.2559(0.0772)	0.0440(0.0830)	-0.7398(0.0712)	0.7471(0.0197)	0.1543
NMG	0.6212(0.1467)	-0.7837(0.1755)	0.3454(0.2143)	1.1601(0.1008)	0.0924
LN	0.4876(0.0565)	-0.3036(0.0745)	-0.1570(0.1324)	0.8904 (0.0449)	0.1702
JL	1.0725(0.2548)	-1.3178(0.4473)	0.9269(0.4898)	1.3111(0.1869)	0.1856
HLJ	0.5717(0.0513)	-0.4042(0.0576)	0.2741(0.1231)	0.9464(0.0230)	0.1450
SH	-0.0396(0.0931)	0.0133(0.0473)	0.0834(0.1609)	1.0215(0.0514)	0.2076
JS	-0.7575(0.1365)	1.5353(0.3535)	-4.1511(1.0696)	0.2741(0.2258)	0.1126
ZJ	0.6758(0.0169)	-0.3349(0.0247)	-0.5429(0.0531)	0.8131(0.0121)	0.1454
AH	0.0840(0.0784)	0.0859(0.0842)	-0.3291(0.0915)	0.8758(0.0366)	0.1974
FJ	0.4849(0.0468)	-0.4049(0.0667)	-0.1745(0.1082)	0.9964(0.0287)	0.1139
JX	1.5093(0.2093)	-1.2774(0.2362)	-0.2192(0.3245)	0.9748(0.1395)	0.0586
SD	0.0381(0.1405)	0.2555(0.2070)	-0.8115(0.4179)	0.7695(0.0912)	0.1345
HN	0.1072(0.0787)	-0.1355(0.0916)	0.0469(0.2892)	1.0313(0.1019)	0.1979
HUBEI	1.2195(0.2260)	-1.1544(0.3354)	0.4008(0.3833)	1.0769(0.1254)	0.2077
HUNAN	-0.0012(0.0984)	0.7778(0.2334)	-1.7958(0.3617)	0.3183(0.1426)	0.1343
GD	0.4026(0.1937)	-0.3351(0.0446)	0.0019(0.3378)	0.9961(0.1350)	0.1878
GX	0.8396(0.1317)	-0.4486(0.2482)	-0.5851(0.2490)	0.7720(0.1243)	0.1817
HAINAN	2.0727(0.3982)	-1.9543(0.0723)	0.5259(0.8458)	1.3886(40.3054)	0.1989
CQ	0.6039(0.0409)	-0.0116(0.0688)	-1.3972(0.2602)	0.5253(0.0868)	0.1462
SC	0.2199(0.1797)	0.2627(0.1480)	-1.0071(0.3783)	0.5907(0.1656)	0.1736
GZ	1.5694(0.2465)	-1.2004(0.2028)	0.4720(0.1618)	0.8989(0.0570)	0.2378
YN	0.0479(0.1126)	-0.0114(0.0573)	-0.2621(0.1044)	0.9571(0.0531)	0.2349
SHANXI	0.6409(0.0983)	-0.6007(0.1049)	0.1889(0.1040)	1.0399(0.0440)	0.1829
GS	1.3141(0.4953)	-0.3095(0.0973)	-1.7702(0.8154)	0.1973(0.3593)	0.1463
QH	1.0271(0.2176)	-0.8680(0.1704)	-0.1624(0.1162)	0.8640(0.0621)	0.0724
NX	0.7751(0.2488)	-0.6075(0.1214)	0.3017(0.2983)	0.9597(0.1559)	0.2294
XJ	0.6339(0.1080)	-0.4303(0.0740)	-0.2491(0.0721)	0.8667(0.0291)	0.2316
Nationwide	0.8906(0.0046)	-0.5851(0.0036)	0.0689(0.0030)	0.5375(0.0017)	27.2653

Standard errors are within parentheses.

Based on the estimation outcomes in Table 4, a Sargan test was performed. The p-value of the J statistic is 0.7238, thus accepting the null hypothesis of no overidentifying restrictions of the model. In addition, an Arellano-Bond test was performed for the dynamic panel data. The p-value was 0.9617, which rules out serial correlation of the residual in the model. Based on these results, the following can be observed:

(1) Taken together, the results indicate that carbon emissions are characterized by inertia. A 1% increase in carbon emissions in the previous year leads to a 0.5375% increase in current carbon emissions, which is consistent with the conclusion reached by Zhang et al. [17]. This result reveals that carbon emissions reduction is a difficult, systematic project. That is, only when the total carbon inhibition generated by various paths in the current year offsets the pulling effect induced by an increase in carbon emissions in the previous year do carbon emissions decline in the current year. Second, *α* and *β* measure the impact of *LGI* on carbon emissions through the structural and efficiency paths, respectively. As observed, a 1% increase in *LGI* can inhibit carbon by 0.8906% and 0.5851% through industrial structure optimization and energy efficiency improvement, respectively, which is consistent with the results of the direct effect model as well as with the focus on supply-side reforms in China. Third, *γ* represents the impact of technical factors (energy efficiency) on carbon emissions by guiding the industrial structure. It plays a very small role of 0.0689, which is inconsistent with the theory. Supported by the estimation results excluding the first-order lag of carbon emissions, we believe that technical factors can play a leading role in China’s industrial structure optimization, but the effect of carbon emissions generated by technical factors through the industrial structure is affected by carbon emissions inertia. This conclusion is similar to that of Zhou et al. [48]. Meanwhile, as Wang et al. indicated, technological progress can promote a low-carbon industrial structure [60]. However, given the number of limiting factors, at the current stage, investment should be used mainly to achieve a low-carbon industrial structure.

(2) Given the inertia of carbon emissions, regional differences in the impact of *LGI* on carbon emissions still exist in the sampled provinces through related paths. In terms of the structural path, with the exception of Jiangsu, Shanghai and Hunan, *LGI* in other provinces can inhibit carbon emissions to varying degrees by guiding the industrial structure. The carbon inhibition coefficients of Hainan, Guizhou, Jiangxi, Gansu, Hubei, Jilin, Qinghai, Beijing, Guangxi, Ningxia, Zhejiang, Shaanxi, Xinjiang, Inner Mongolia and Chongqing are all above 0.6, while they are below 0.1 in Shandong, Yunnan, Hebei and Anhui. In practice, although the level of economic development in Tianjin, Jiangsu and Zhejiang, which are located in the eastern area, is relatively high, industrial structure optimization within the sample time period is not obvious; thus, the guiding role of *LGI* in the industrial structure has not been fully revealed. In terms of the efficiency path, with the exception of Shanghai, Shanxi, Anhui, Shandong, Sichuan, Hunan and Jiangsu, *LGI* can inhibit carbon emissions by guiding energy efficiency to varying degrees. The carbon inhibition coefficients of Hainan, Jilin, Jiangxi, Guizhou, Hubei, Qinghai, Inner Mongolia, Ningxia and Shaanxi all exceed 0.6, while they are below 0.1 in Chongqing and Yunnan. The emissions reduction effect of the efficiency path is highest in the western area, followed by the northeast area. The emissions reduction effect is relatively small in the central and eastern areas, indicating that there is considerable room for *LGI* to guide energy efficiency (technical) aspects in the central and eastern areas. For Jiangsu, Shanghai and Hunan, *LGI* does not inhibit carbon emissions through the related paths. On the contrary, it generates a weak carbon pulling effect. In practice, Jiangsu, Shanghai and Hunan are major energy consumers in China, and the rapid economic growth in those provinces leads to constant increases in energy consumption. As a result, *CO*_*2*_ emissions increase yearly. The inertia that is characteristic of carbon emissions may result from the fact that it is difficult for the carbon emissions reduction effect generated by *LGI* to offset the carbon pulling effects of economic growth and inertia. Furthermore, other than in Guangdong, Henan, Shanghai, Shaanxi, Heilongjiang, Ningxia, Inner Mongolia, Hubei, Hebei, Guizhou, Hainan and Jilin, technical factors (energy efficiency) in the other provinces can inhibit emissions to a certain extent by guiding the industrial structure, possibly because these provinces have experienced rather small changes in the industrial structure and thus did not fully demonstrate the guiding role of technical factors during the study period.

(3) There are evident regional differences in the carbon emissions reduction generated by *LGI*. The estimation results of the static and dynamic models show that the carbon inhibition of *LGI* through the related paths is most significant in the western area. In the static model, the carbon inhibition effects of the four areas in descending order are western area > northeast area > eastern area > central area. However, in the dynamic model, the corresponding effects are western area > central area > eastern area > northeast area. These results illustrate that in the central and eastern areas, a strong carbon emissions reduction effect in the previous year to a certain extent magnifies the policy effect in the current year due to carbon emissions inertia.

## Regional differences in the carbon emissions reduction effect of LGI

**Fig 2:**
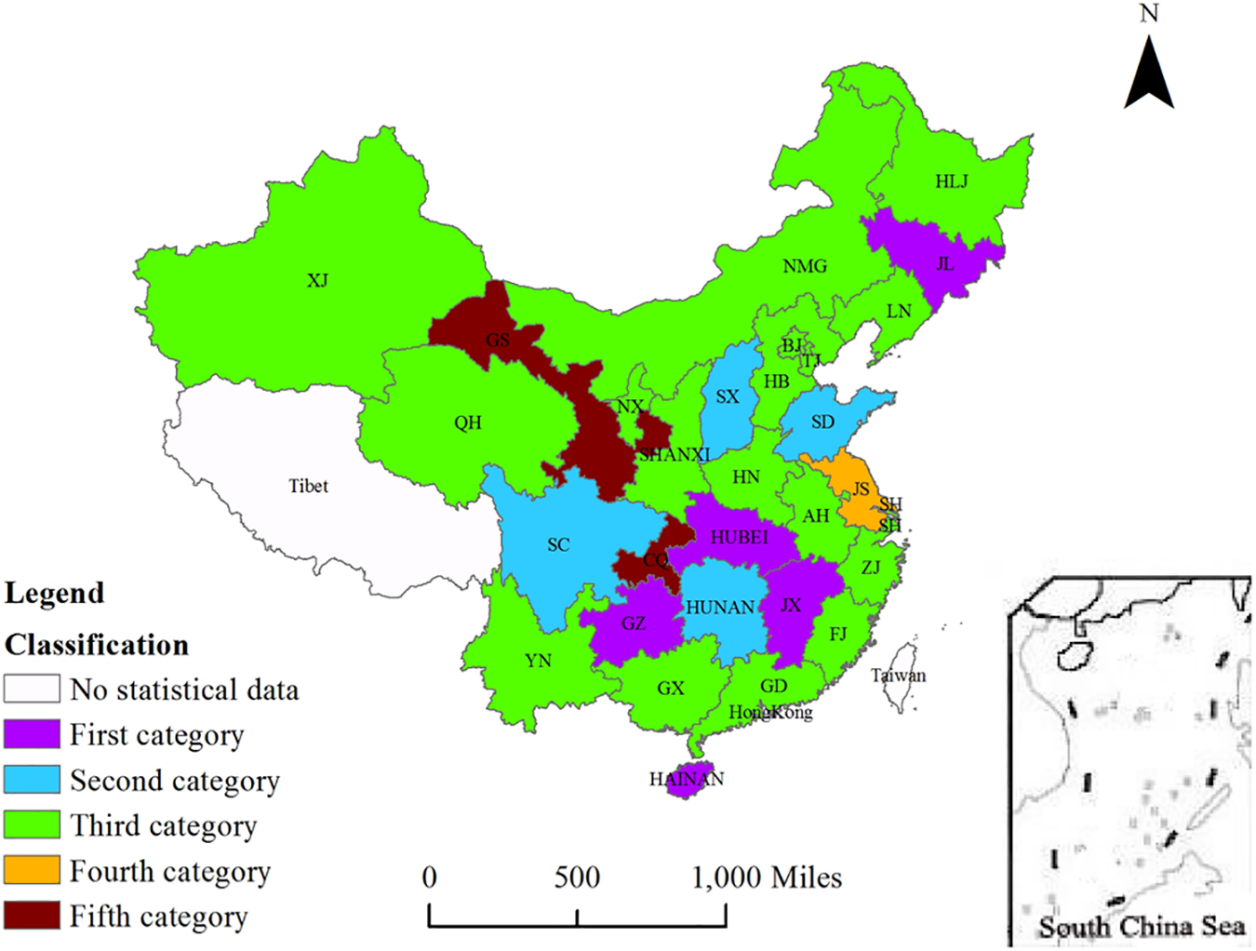
Regional categorization of carbon emissions inhibition by *LGI*.

Differing economic development levels, investment structures and policy orientations result in differences in the carbon emissions reduction effects of government investment. To further explore the corresponding patterns, the 30 sampled provinces are divided into groups based on the estimation results of the dynamic model. This study adopts the K-means clustering method to classify them into five categories. The results are shown in Fig 2.

The five categories are as follows: in the first category (Jilin, Jiangxi, Hubei, Hainan and Guizhou), *LGI* generates strong carbon inhibition through the related paths; in the second category (Shanxi, Shandong, Hunan and Sichuan), carbon inhibition from *LGI* is weak; in the third category (Beijing, Tianjin, Hebei, Inner Mongolia, Liaoning, Heilongjiang, Shanghai, Zhejiang, Anhui, Fujian, Henan, Guangdong, Guangxi, Yunnan, Shaanxi, Qinghai, Ningxia and Xinjiang), carbon inhibition is relatively weak; in the fourth category (Jiangsu), carbon inhibition is medium strength; and in the fifth category (Chongqing and Gansu), carbon inhibition is relatively strong.

Overall, (1) the carbon emissions reduction effect of *LGI* is strongest in Hainan. In recent years, Hainan has increased its investment in energy-saving technology projects. The pilot projects of some key enterprises in the building, agriculture, fishing and other industries have effectively enhanced energy saving and reduced emissions. (2) There is no direct correlation between the carbon emissions reduction effect of *LGI* and the absolute level of each area’s economic development. Each category, especially the third category, includes provinces of varying levels of economic development. The impact of *LGI* on carbon emissions might be related to geographic location, provincial carbon emissions levels and regional policies that are designed to reduce emissions. In some provinces in the western, central and eastern areas, *LGI* shows relatively significant carbon inhibition. However, the study by Shen et al. shows that the effects of energy saving and emissions reduction implemented by China’s local governments are mainly related to the economic development level [36]. The top ten provinces are mainly in the east, where the level of economic development is high; thus, on the one hand, their economies have a rather solid basis with sufficient financing to buttress the implementation of energy conservation and emissions reduction policies. On the other hand, as their economies have moved into the critical period of strategic transformation, the local governments have paid much more attention to energy conservation and emissions reduction. That conclusion is not consistent with this study, which maintains that in recent years and under the guidance of uniform national planning, Chinese provinces have all formulated their own targets for energy conservation and emissions reduction and have paid considerable attention to the environmental benefits. Local governments in the eastern area do not pay more attention to environmental protection than do local governments in the central and western areas. Second, although the economic development level in the eastern area is relatively high, thus creating a comparative financial advantage, the Chinese government has long set preferential policies targeted at the western area due to the specific conditions there. Financial investment in energy conservation and emissions reduction in the western area, such as in Gansu and Shanxi, is also rapidly increasing. Third, compared with the central and western areas, the eastern area has a better industrial structure and a higher technical level. Thus, it is difficult for investment to guide the industrial structure and technical efficiency. By contrast, the industrial structure in the western area is less developed and the technical level is quite low, which means that policy guidance is much more evident. Therefore, this study shows that the preferential policies implemented by the Chinese government in the western area have led to an emphasis on the development of industries with local advantages through preferential fiscal, monetary, price and tax policies, which has facilitated the optimization of the industrial structure and thereby magnified the carbon emissions reduction effect of *LGI*. In the central area, the emissions reduction effects generated by *LGI* are relatively high. In practice, with the full implementation of the “Rise of Central China Plan” (The Rise of Central China Plan refers to a policy implemented by the central government to facilitate the development of China’s central economic zone, including Henan, Hubei, Hunan, Jiangxi, Anhui and Shanxi. It was first proposed by Premier Wen Jiabao on March 5, 2004), *LGI* in the central area has been optimized and adjusted to the local industrial structure of high-efficiency and low-energy-consumption products, which, combined with energy conservation and emissions reduction initiatives, has led to a significant reduction in emissions. In recent years, Beijing and Tianjin, which are in the eastern area, have also increased their investment in energy conservation and emissions reduction, which has led to industrial structure optimization, technological innovation and improvements in energy efficiency, consequently reducing emissions. Although the old industrial base in the northeast area is increasing the pace of the transformation of and upgrades to its industrial structure, in the short term, it is unlikely to improve significantly. Zhou notes that China’s *LGI* is at an investment competition stage [61], such that it relies heavily on secondary industry and provides little impetus to tertiary industry, thereby remaining unlikely to adjust the industrial structure. This argument conforms to the actual conditions in the northeast area. In addition, some enterprises in the northeast still have no emissions control facilities and directly contribute to air pollution. Coal-fired heating in the winter also increases carbon emissions. The results of this empirical study show that carbon emissions are characterized by inertia, and high levels of carbon emissions offset reductions from related paths. For this reason, it is difficult for the guiding role of *LGI* to manifest itself. As one can see, the structure of the regional industry, efficient levels of energy use, carbon emissions levels and policy orientation all influence the effect of *LGI* on carbon emissions.

## Conclusions

This study measures the direct and regulating role of *LGI* on carbon emissions in 30 sampled provinces in China and categorizes these provinces using a dynamic regulating model. First, the empirical study concluded that within the sample time period, *LGI* can inhibit carbon emissions by guiding the industrial structure and energy efficiency. The role of the structural path is stronger than that of the efficiency path. Second, when carbon emissions inertia is considered, the carbon emissions reduction effects of *LGI* are improved to some extent through these two paths, which indicates that good carbon emissions reduction effects magnify the carbon inhibition generated by *LGI*. Meanwhile, in places with sizeable carbon emissions, carbon inhibition through *LGI* might be offset by carbon emissions inertia; therefore, its role is hardly evident. Third, there are regional differences in the impact of *LGI* on carbon emissions. For most provinces in China, *LGI* can generate an effective carbon inhibition effect. However, for some places, such as Jiangsu, Shanghai and Hunan, *LGI* does not generate effective carbon inhibition through the related paths. Instead, it generates a carbon pulling effect, which indicates that economic growth in these places has not yet achieved carbon decoupling. Fourth, based on the dynamic comprehensive regulating effect of *LGI* on carbon emissions, the 30 sampled provinces are classified into five categories. *LGI*’s role in carbon emissions reduction is classified into the categories of strong, relatively strong, medium, relatively weak and weak. On the whole, the carbon emissions reduction effect of *LGI* has no apparent correlation with the local economic development level. Instead, it is influenced by industrial structure characteristics, efficient energy use, historical carbon emissions levels and policy directions. Relatively speaking, the carbon emissions reduction effects in the western and central areas of China are more evident. It is difficult for *LGI* in most provinces in the eastern and northeast areas to effectively inhibit carbon emissions. Furthermore, as noted in Section 2, there is a theoretical inverted U-shaped relationship between *LGI* and carbon emissions. The results of this empirical study reveal that for some provinces, the carbon pulling effect of increases in economic aggregates as a result of *LGI* exceeds the carbon inhibition generated through the structural and efficiency paths and therefore does not generate emissions reduction effects. For other provinces, the carbon inhibition generated by *LGI* through these two paths effectively offsets the carbon pulling effect induced by investment to promote economic growth. This difference also reflects the linkage between the investment structure and carbon emissions to some degree. In actual economic operations, reducing carbon emissions is a goal that every local government pursues while attempting to realize economic growth. According to this study, this goal can be achieved through structural optimization, improvements to efficiency and policy regulation and guidance, which is consistent with the core idea emphasized in China’s “new normal” economy.

In practice, more attention should be paid to the role of *LGI* in guiding the industrial structure and technological innovation in order to achieve economic growth with environmental benefits. In practice, the investment structure should be optimized to improve investment efficiency. The outline of the Thirteenth Five-year Plan for National Economic and Social Development of the People's Republic of China (hereafter, the Thirteenth Five-year Plan) clearly states that local governments should base their investments on effective demand to optimize the supply structure and increase investment efficiency; furthermore, it emphasizes that the government should make investment a fully leveraged role. On the other hand, coordination among investment, industrial policies, technological innovation, energy strategies and other emissions reduction policies should also be emphasized. Similarly, the Thirteenth Five-year Plan should consider the improvement of environmental quality as a core objective in addition to structural optimization and increasing the efficient use of resources. The results of this empirical study show that the carbon emissions reduction effect of *LGI* is subject to the local industrial structure, the level of energy consumption, energy use efficiency and other factors. Coordination among multiple policies can magnify the effect of investments to reduce carbon emissions. Furthermore, there is significant regional diversity in the impact of *LGI* on carbon emissions. Therefore, the spatial distribution of *LGI* should also be coordinated.

In terms of policy focus, the economic gap between regions should be further narrowed to achieve balanced development. Meanwhile, cross-regional environmental protection agencies should be established to promote the formation of regional and cross-regional policy systems. Additionally, local governments should assess their own situations, follow the evolutionary laws of the industrial structure, further optimize their industrial structures and adjust their investment strategies and orientations to fit local conditions. In order to promote comprehensive energy conservation, local governments should improve their provinces’ efficient energy use through effective policies and technologies and work toward multi-level coordination among *LGI*, industrial, scientific and technological policies, and other macro regulation and control measures, such as price and taxation policies.
